# Spatial heterogeneity of cutaneous blood flow respiratory-related oscillations quantified via laser speckle contrast imaging

**DOI:** 10.1371/journal.pone.0252296

**Published:** 2021-05-27

**Authors:** Irina Mizeva, Elena Potapova, Viktor Dremin, Igor Kozlov, Andrey Dunaev

**Affiliations:** 1 Institute of Continuous Media Mechanics UrB RAS, Perm, Russia; 2 Research & Development Center of Biomedical Photonics, Orel State University, Orel, Russia; 3 College of Engineering and Physical Sciences, Aston University, Birmingham, United Kingdom; University of Illinois at Urbana-Champaign, UNITED STATES

## Abstract

LSCI technique provides experimental data which can be considered in the context of spatial blood flow coherency. Analysis of vascular tone oscillations gives additional information to ensure a better understanding of the mechanisms affecting microvascular physiology. The oscillations with different frequencies are due to different physiological mechanisms. The reasons for the generation of peripheral blood flow oscillations in the 0.14–0.6 Hz frequency band are as follows: cardio-respiratory interactions, pressure variations in the venous part of the circulatory system, and the effect of the sympathetic nervous system on the vascular tone. Earlier, we described the spatial heterogeneity of around 0.3 Hz oscillations and this motivated us to continue the research to find the conditions for the occurrence of spatial phase synchronization. For this purpose, a number of physiological tests (controlled respiration, breath holder, and venous occlusion tests) which influence the blood flow oscillations of 0.14–0.6 Hz were considered, an appropriate measurement system and the required data processing algorithms were developed. At spontaneous respiration, the oscillations with frequencies around 0.3 Hz were stochastic, whereas all the performed tests induced an increase in spatial coherence. The protocols and methods proposed here can help to clarify whether the heterogeneity of respiratory-related blood flow oscillations exists on the skin surface.

## Introduction

The study of vascular tone oscillations is needed to gain deeper insight into the mechanisms or factors that affect microvascular physiology. In the spectra of peripheral blood flow, there are several peaks which correspond to different physiological mechanisms of vascular tone regulation [1]. The oscillations with frequencies close to 1 Hz are caused by the activity of the heart and are related to pulse wave propagation. The characteristic time of the arteriole wall muscle activity is approximately 10 s, and the modulation (0.1 Hz) is observed in the peripheral blood flow signals [2].

The blood flow oscillations with frequency 0.145–0.6 Hz is associated with the respiration [3] It is widely accepted now that the respiratory-related modulation of the peripheral blood flow is caused by the intrathoracic pressure, which determines the dynamics of the breathing pump (blood venous return to the heart from the periphery) [4, 5].

Cardiorespiratory coupling [6] and its variation due to age-related [7] and pathological factors have received much consideration. Different tests, including control breathing trials, were applied to study respiratory-related blood flow oscillations in the microcirculatory system [8, 9]. In Refs. [10, 11], an attempt was made to determine a possible weakening of the time-phase relationship between the breathing rhythm and the peripheral pulse, which was measured with Laser Doppler flowmetry (LDF). It was shown that Type 1 Diabetes and this weakening can be the risk factors for diabetic microangiopathy.

Physiological studies on microcirculation are most commonly performed on the distal part of the extremities. To analyze the spatial distribution of the microcirculation, it is convenient to use the palm with a large enough flat surface and well vascularization in the upper layers of the skin [12]. Blood flow oscillations can be quantified by LDF [13, 14], temperature measurements [15], photoplethysmography [16], high-speed videocapillaroscopy [17], as well as by laser speckle contrast imaging (LSCI) [18]. The high correlation between the LSCI and LDF methods predicted by Fredriksson in Ref. [19] was confirmed experimentally in Ref. [20]. In Ref. [20], we demonstrated for the first time the possibility of detailed spectral analysis of laser speckle contrast signals, which made it possible to study the spatial variations of blood flow oscillations.

LSCI is a simple and powerful tool for determining the spatial distribution of the perfusion with high temporal resolution [21]. Normal skin perfusion is non-uniformly distributed at the surface [22, 23]. As described earlier [20], we have observed that the respiratory-related oscillations vary in phase and amplitude from point to point on the skin surface. In this work, we search for the tests in which not only the energy of the respiratory-associated peripheral blood flow oscillations can be evaluated but also their spatial distribution. The work is based on the preliminary presented measurements [24]. In this study, we extend physiological tests for assessing respiratory-associated oscillations in peripheral vessels by applying a venous occlusion test. Additionally, we have developed a series of surrogate tests to provide statistical significance estimate for parallel arrays and to test the validity of the experimental data obtained.

Analysis of the spatial coupling of blood flow oscillations described in Refs. [25, 26] has indicated that physiological tests can vary the coherence of peripheral blood flow at different points. To measure the oscillations with different frequencies, an impact spectral analysis is utilized. For the short nonstationary data obtained using wavelets [27], the efficiency of this analysis has been proved in Refs. [1, 28]. This method allows one to study the phase relations between oscillations with different frequencies and to perform the coherence analysis. Estimation of the spatio-temporal spectral analysis as far as the correlation analysis of the dynamics of perfusion of the surface, still remains a challenging task. The reliability of the results is of particular importance. To evaluate the obtained coherence, surrogate techniques are commonly applied [29–31].

Surrogate methods usually produce artificial data by randomizing the property to test but mimicking other properties (e.g., the spectra) of the original signal as much as possible [32]. In our opinion, the coupling analysis methods for multidimensional data have not yet been developed; we could not find any publications on this topic in the literature.

The aim of this work is to study the spatial distribution of respiratory-related blood flow oscillations on the skin surface using the original LSCI signal processing technique. The main hypothesis of this work is that “switching off” respiration can increase the synchronization of the blood flow oscillations in the respiratory range. Section **Physiological tests** discusses the variety of physiological tests used in the research. Section **Materials and methods** describes the experimental setup, study protocols, and calculation methods. Further the obtained **Results** are presented. In the **Discussion** the hypothesis for the spatial heterogeneity of the blood flow respiratory-related oscillations with frequencies close to the respiration frequency is formulated.

## Physiological tests

At present, there are various physiological tests intended to detect changes in microvascular function [33, 34]. Let us consider the tests in which the effect of respiration on the venous circulation is investigated. All these tests have indirect influences on the blood flow in the peripheral vessels.

The location of data collection is the palmar surface area, where perfusion is usually quite high and the capillary network is dense. Besides, this skin region is rich with arteriovenous anastomoses and has well-developed adrenergic innervation.

Controlled respiration test (**CRT**) [35] is widely used in studies on cardio-respiratory coupling [36]. The cardiovascular and respiratory autonomic efferent fibers have a common central origin [37]. Variations in respiration influence cardiac and vascular function through the modulation of neural circulatory control mechanisms [38]. The use of different types of breathing can affect the value of sympathetic-vagal balance [39]. In Ref. [8], respiration tests with different breath rates are utilized to investigate various cardiovascular couplings observed in the signals. Significant changes of coherency of cardiovascular signals are obtained.

Breath holding test (**BHT**) is used to study the active vasoconstrictor mechanisms. The increased impulses from the carotid glomus lead to a number of changes in the status of the autonomic nervous system and regulation of the respiratory and cardiovascular systems. The result is an increase in the tone of the sympathetic nervous system and inhibition of the baroreflex regulation of the cardiovascular system [40]. The vasomotor reflex leads to an impermanent decrease in blood perfusion in response to deep and rapid inspiration [41].

Venous occlusion test (**VOT**) induces constriction of the supplying arterioles and, consequently, an increase in precapillary resistance [42, 43]. The precapillary constriction mainly occurs due to the activation of a veno-arteriolar reflex that is mediated by sympathetic postganglionic fibers without requiring transmission through the spinal cord [42, 44]. Partly, constriction is caused by the myogenic response and the rise in intravascular pressure, or by a direct damming back effect of venous blood and obstruction of capillary outflow [45, 46]. The use of a venous occlusion test for studying respiratory oscillations makes it possible to switch off the respiration pump and to detect changes in blood flow taking into account local factors.

## Materials and methods

### Experimental study

**Fig 1:**
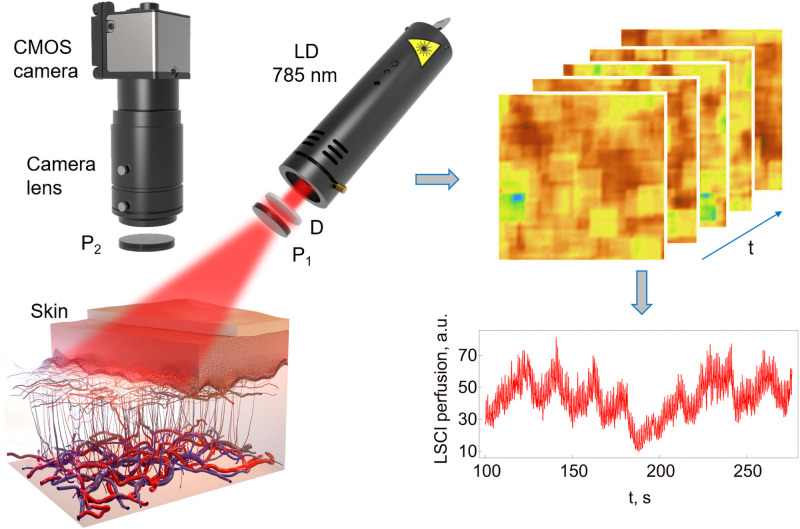
Scheme of the experimental setup and the LSCI pattern plotted using the results averaged over the whole image. LD—laser diode, P_1_ and P_2_—linear polarizers, D—diffuser.

The scheme of the experimental setup is presented in Fig 1. The area under study was illuminated by a 10 mW laser source operating at 785 nm wavelength (Thorlabs Inc, USA). A CMOS camera DCC3260M (Thorlabs, Inc., USA) with 1936 × 1216 pixels and 5.86 *μ*m pixel size, camera lens MVL25TM23 (Thorlabs, Inc., USA) and a diffuser for uniform illumination of the area of interest were used to record raw speckle images. The illumination channel and camera were equipped with linear polarizers fixed at the crossed position for the reduction of specular reflection from the tissue surface. The data was acquired at the sampling frequency of 80 Hz and an exposure time of 9 ms. The distance between the camera lens and the examined area was 25 cm.

The typical raw speckle map is shown in Fig 1. For maximization of the signal-to-noise ratio, the minimal speckle size must exceed the Nyquist criterion [47]. Thus, the speckle size on the camera was adjusted by changing the pupil diameter of the camera lens to achieve a speckle size at least 2 times the pixel size. We attempted to avoid all movements and external vibrations. The hand was placed on the pneumatic vibration isolation workstation (1VIS10W, Standa, Lithuania) with the optical setup. The hand was thermally isolated from the table and additionally secured by gentle wrapping with an elastic cohesive bandaging material “Peha-haft” (Hartmann, Germany) to prevent involuntary movements.

**Fig 2:**
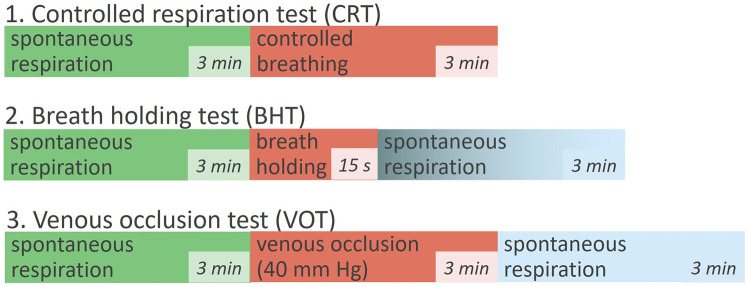
Scheme of three experimental protocols: Controlled respiration test (CRT), breath holding test (BHT), venous occlusion test (VOT).

Our preliminary study included only one subject (female, 37 years old, without cardiovascular disease, nonsmoker). The data was collected from the base of the palm (hypothenar eminence). Fig 2 shows the three different protocols used in the study. In all tests, spontaneous respiration (**SR**) was applied as a reference. The respiratory rate was set by the metronome when conducting **CRT**. During the BHT deep and rapid maximal inspiration was used. The applied cuff pressure was 40 mmHg during **VOT**. The study was approved by the Orel State University Ethics Committee (protocol No.15, 21.02.2019).

### Data analysis

To perform data analysis, we cropped full screen images to the ROI with 400 × 400 pixels, approximately 8 × 8 mm. We calculated the speckle contrast over the whole ROI and get the spatio-temporal perfusion dynamics. Then we split the ROI on 64 *roi*_*i*,*j*_ and reconstructed temporal dynamics *f*_*i*,*j*(*t*)_ for every *roi*_*i*,*j*_ averaging by space. This size of single *roi*_*i*,*j*_ was chosen because it is close to the typical size of the microvascular unit which is supplied by one ascending arteriole [48] The algorithm is described in detail in Ref. [20].

The images obtained were processed with a custom-developed algorithm using Matlab R2018b. The average speckle contrast of the image was calculated as [21]

$$\begin{array}{c} K=\sigma_{N}/{\left\langle I_{0}\right\rangle }_{N}, \end{array}$$ (1)

where 〈〉 is the symbol of averaging, *N* is the window of averaging *N* × *N*, and *σ*_*N*_ is the standard deviation in the window *N* × *N* where *N* is equal 7.

Furthermore, wavelet decomposition is applied to each *f*_(*i*,*j*)_ using algorithms based on the Morlet wavelet [27].

$$\begin{array}{c} \psi \left(t\right)=e^{2\pi it}e^{-t^{2}/2\sigma^{2}}. \end{array}$$ (2)

The decay parameter *σ* = 1.7 defines the length of the analyzing function (the number of oscillation) [20] and *t* is time.

The continuous wavelet transform of the signal *f*_*i*,*j*_(*t*) is determined as

$$\begin{array}{c} W_{i,j}\left(\nu ,\tau \right)=\sqrt{\nu }\int_{-\infty }^{\infty }f_{i,j}\left(t\right)\psi^{*}(\nu (t-\tau ))dt, \end{array}$$ (3)

where * means complex conjugation. The wavelet decomposition of 1D data returns a 2D array of complex values which can be represented by their modulus |*W*_*i*,*j*_(*ν*, *τ*)| [49]:

$$\begin{array}{c} |W_{i,j}\left(\nu ,\tau \right)|=\sqrt{Re{\left(W_{i,j}\left(\nu ,\tau \right)\right)}^{2}+Im{\left(W_{i,j}\left(\nu ,\tau \right)\right)}^{2}} \end{array}$$ (4)

and their phase *ϕ*_*i*,*j*_(*ν*, *τ*)

$$\begin{array}{c} \phi_{i,j}\left(\nu ,\tau \right)=\text{arctan}\frac{Im(W_{i,j}\left(\nu ,\tau \right))}{Re(W_{i,j}\left(\nu ,\tau \right))}. \end{array}$$ (5)

Upon integrating the power with respect to time, we get the global wavelet spectrum

$$\begin{array}{c} M_{i,j}\left(\nu \right)=\frac{1}{T}\int_{0}^{T}{\left|W_{i,j}\left(\nu ,\tau \right)\right|}^{2}d\tau , \end{array}$$ (6)

which is the analogue of the Fourier spectrum (power spectral density) [15].

The extracted phase is associated with each frequency and time, and it forms the basis of phase coherence. The phase difference between two signals remains unchanged with time at each frequency. The coherence is estimated as [50]

$$\begin{array}{c} C_{i,j,l,m}=\sqrt{{\left\langle \text{cos}\Delta \phi_{i,j,l,m}\left(\nu ,\tau \right)\right\rangle }^{2}+\langle \text{sin}\Delta \phi_{i,j,l,m}{\left(\nu ,\tau \right)\rangle }^{2}}, \end{array}$$ (7)

where Δ*ϕ*_*i*,*j*,*l*,*m*_(*ν*, *t*) = *ϕ*_*i*,*j*_(*ν*, *t*) − *ϕ*_*l*,*m*_(*ν*, *t*), is the instantaneous phase of *f*_*i*,*j*_(*t*) and *f*_*l*,*m*_(*t*) signals. If the phase difference is almost constant, the phase coherence tends to become 1 [51], if it Δ*ϕ*_*i*,*j*,*l*,*m*_(*ν*, *t*) is random at a given *ν*, then *C*_*i*,*j*,*l*,*m*_ tends to zero.

In this work, we calculated *W*_*i*,*j*_(*ν*, *τ*) for every *f*_*i*,*j*_(*t*). The frequency range of 0.07–2 Hz was split logarithmically into 40 frequency bands. The power spectrum was averaged over all 64 *roi*. Finding the coherency for all possible pairs presents difficulties because it requires a lot of calculations. To this end, we have chosen 100 pairs (*i*, *j*), calculated coherency, and then averaged the data obtained from the sample. It has been established that the sample of 100 randomly selected pairs gives full statistics, and a further increase in the number of pairs does not change the result. All statistical characteristics presented in the paper were computed using Mathematica 9.0 (Wolfram, USA).

### Surrogate data calculations

The results of the wavelet phase coherence analysis should be verified using surrogate techniques [29]. Most surrogate techniques assume a Gaussian distribution of the underlying process and are presented for low-dimensional data. We deal here with the multidimensional data (64 parallel 1D samples). Using phase-shuffled surrogate techniques [29, 32, 52] for big multidimensional data processing is extremely resource consuming. In this work, we propose two different algorithms for *short* and *long* tests.

The idea of *long* tests accepted here is to use the array of multidimensional data as a source of surrogate data. We shift every *f*_*i*,*j*(*t*)_ to a random Δ*t* and use periodical boundary conditions for the time—so the end of the signal, equal to Δ*t*, is added to the beginning of the signal. Providing such operation, we keep the spectral composition of the signal but vary the phase shift between the signals. The signals become independent from each other. We apply this algorithm to the experimental data to calculate spectral characteristics and wavelet coherency. The approach proposed is suitable for **CRT** and **VOT**, because these tests are sufficiently long.

The *short* **BHT** lasts 15 s, and its time duration cannot be increased significantly. Thus, we have very poor statistics (5 independent oscillations with the frequency of 0.3 Hz). The wavelet with *σ* = 1.7 includes 3 oscillations. It is practically impossible to shift one signal with respect to another, to lose their coupling and to obtain independent surrogate data. In the short test, the technique described in [29] was used. It is interesting that, on the one hand, the boundary effect is rather strong in such short records, but, on the other hand, excluding cycles near the boundaries, it yields extremely low statistics. To reduce the boundary effect, we first calculate the wavelet coefficients of the total signal, then extract the coefficients only in the time interval of interest, and finally evaluate the obtained spectra. For all 64 temporal records, we provide 100 surrogates. For every randomly chosen pair, we estimate the coherency (*C*_*i*,*j*_) and find it for each surrogate pair ($C_{i,j}^{s}$). At a given frequency, we obtain the distribution of coherence values (100 numbers) for each pair. By estimating 0.95 quantile of this distribution, we compare *C*_*i*,*j*_ and 0.95 quantile $C_{i,j}^{s}$. For $C_{i,j}>C_{i,j}^{s}$, assuming that the coherency obtained is significant. Then we calculate the number of pairs with statistically significant coherency (*N*_*significant*_) and compare it with the total number of pairs (*N*_*total*_) under consideration. The ratio of these numbers

$$\begin{array}{c} Q=\frac{N_{significant}}{N_{total}}100\% \end{array}$$ (8)

is part of statistically significant tests.

## Results

### Controlled respiration test

**Fig 3:**
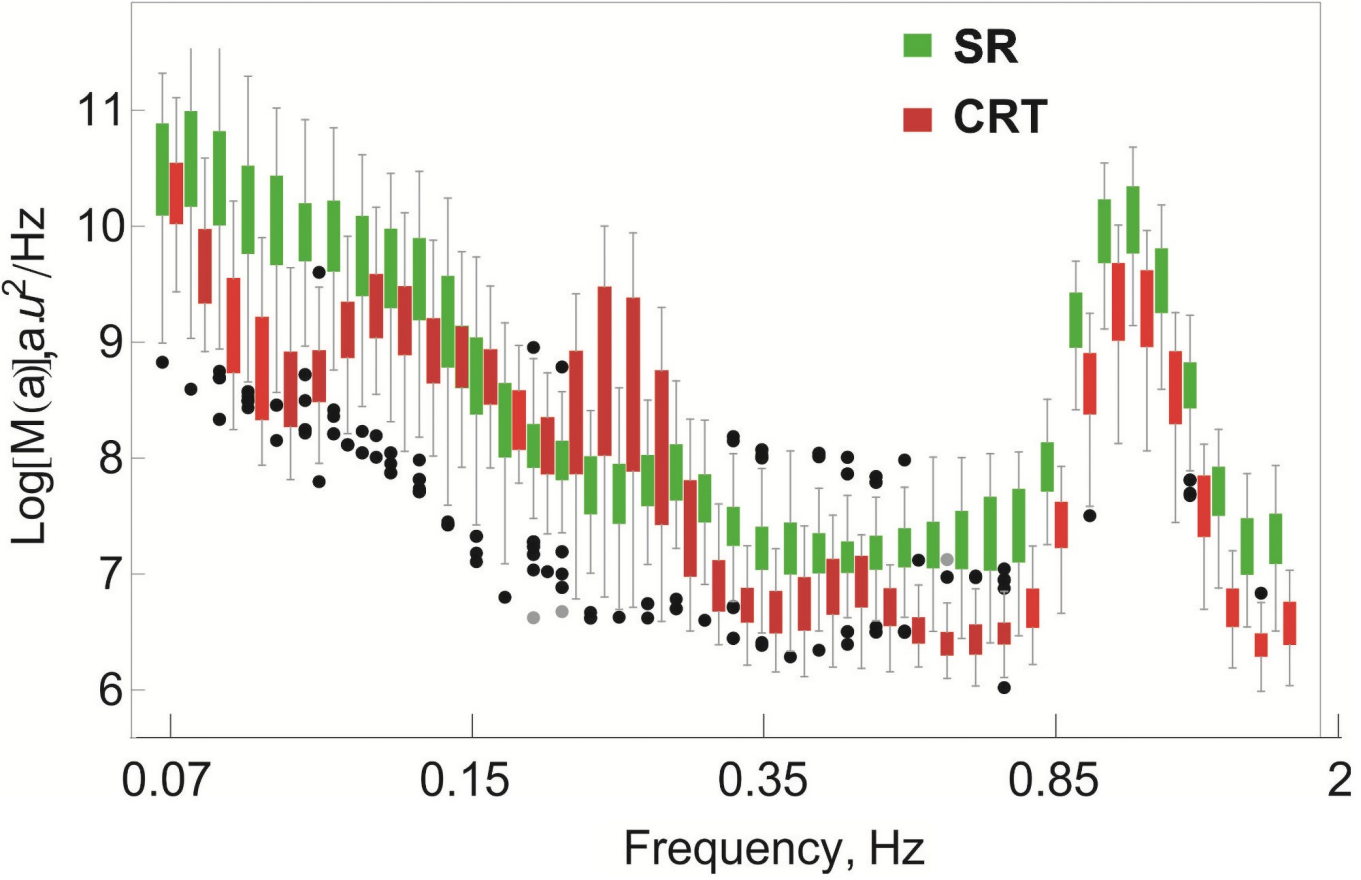
Wavelet spectra averaged over different *roi*, green colour indicate data obtained during SR, red—CRT. Dots indicate outliers.

Comparison of wavelet spectra *M*(*a*) of LSCI temporal signal during **SR** and **CRT** records are shown in Fig 3. Green bars correspond to **SR** and red bars to **CRT**. The controlled respiration frequency is slightly shifted from the mean frequency of the spontaneous respiration, but this deviation is smaller in the right panel and can be attributed to the drawbacks of the experimental protocol.

We measured the median energy of the most powerful oscillations in the respiratory range in **SR** and **CRT** records. It varies from *Log*(*M*(0.3))_*SR*_ = 7.8 to *Log*(*M*(0.3))_*CRT*_ = 8.6.

**Fig 4:**
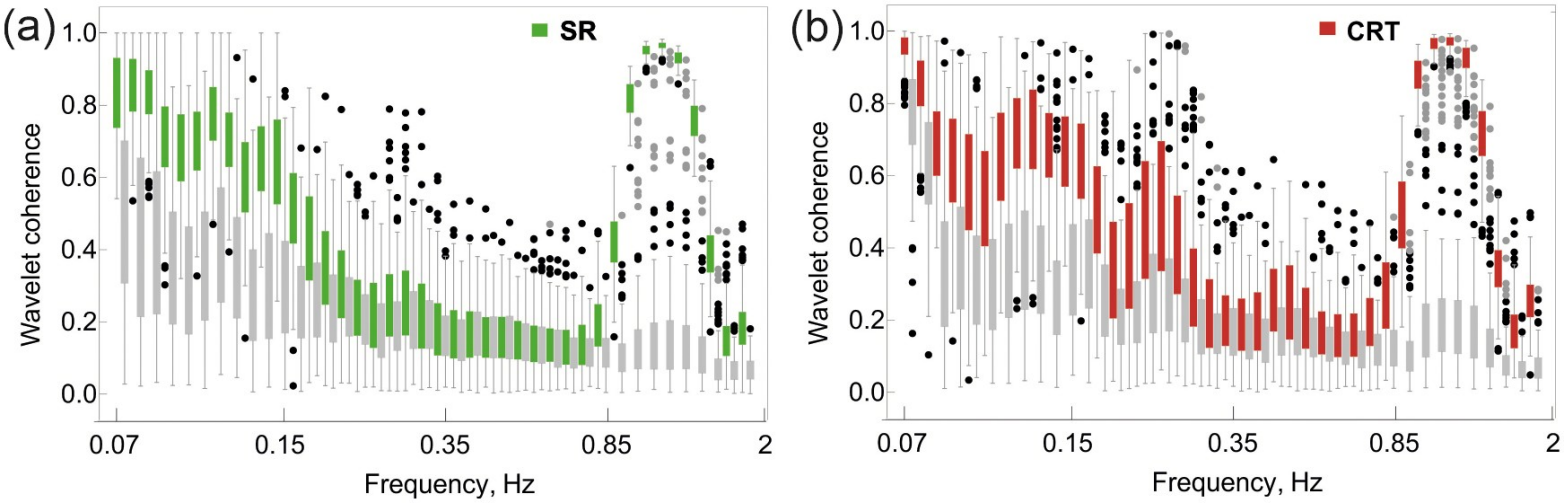
Box whisker diagram describes the wavelet coherence of oscillation of blood flow compared with the surrogate data (plotted by gray) during SR (a) and CRT (b). Dots indicate outliers.

In Fig 4, the wavelet coherence of peripheral blood flow oscillations in **SR** (a) and **CRT** (b) (light and dark green bars) is compared with surrogates (grey bars).

It is obvious that the cardiac-related oscillations are highly correlated in both tests. The same is true for low frequencies in the range 0.07–0.15 Hz. Respiratory-related oscillations in the **SR** are weakly synchronised, and the level of coherency is close to the surrogate data. When the respiration is controlled (**CRT**), the coherency of 0.5 is observed; it is higher than the data observed in surrogates (Fig 4(b)).

### Breath holding test

**Fig 5:**
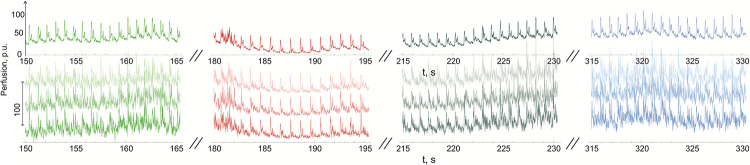
Samples of LSCI perfusion averaged over the total ROI upper curves, and three random *roi* (3 lower curves) in 4 time intervals. Green curves—LSCI perfusion before the **BHT**, red—during the **BHT**, gray—transitional period, blue—recovered perfusion 1.5 min after the **BHT**.

**BHT** manoeuvre allows to exclude the respiration influence. The samples of LSCI averaged over the whole ROI and smaller areas (*roi*) are presented in Fig 5. The upper bright curves correspond to averaging LSCI over the whole ROI and lower, varying in brightness are averaged over the fragments over the ROI. We have chosen 4 characteristic time intervals of the same duration (15 s) before the **BHT** (150–165 s) manoeuvre (green lines), during the **BHT** (180–195 s) (red lines) and two intervals after it: transitional time interval (215–230 s)—gray lines, and 1.5 min after finishing (315–330 s)—blue lines (the system is recovered).

By observing the upper curves obtained by averaging over the whole ROI, we have to bear in mind that during the **BHT** the perfusion becomes lower and therefore this manoeuvre induces vasoconstriction. Secondly, the **BHT** influences the form of the pulse wave. The amplitude of the cutaneous pulse wave becomes lower and the form of the diastolic part of the wave varies significantly. The second peak of the cardiac wave becomes smaller.

The behaviour of the perfusion obtained by averaging the LSCI in *roi* also has its own peculiarities (lower curves in Fig 5). On eye inspection, we found that the perfusion during spontaneous respiration is rather complicated both in time and space. The high frequency noise is present in *roi* temporal records, and it is interesting that the noise level increases during the transitional period (see 215–230 s interval at the beginning and end of the plot).

Furthermore, in all calculations, we process every perfusion data obtained using *roi* and the average characteristics obtained. For spectral characteristics we use all parallel data, and for coherence only 100 random pairs of them. Such an approach allows us to calculate not only the mean characteristics but to estimate their statistical properties. In the respiratory frequency band, we observe a large width of the distribution function of spectral energy, which is indicated as a wide gray corridor. Thus, there is a significant divergence of the LSCI spectra collected from different points. During **BHT**, the signals become more ordered, the corridor along the red curve becomes narrow. After going back to spontaneous respiration, we observe the transitional period and recovery.

**Fig 6:**
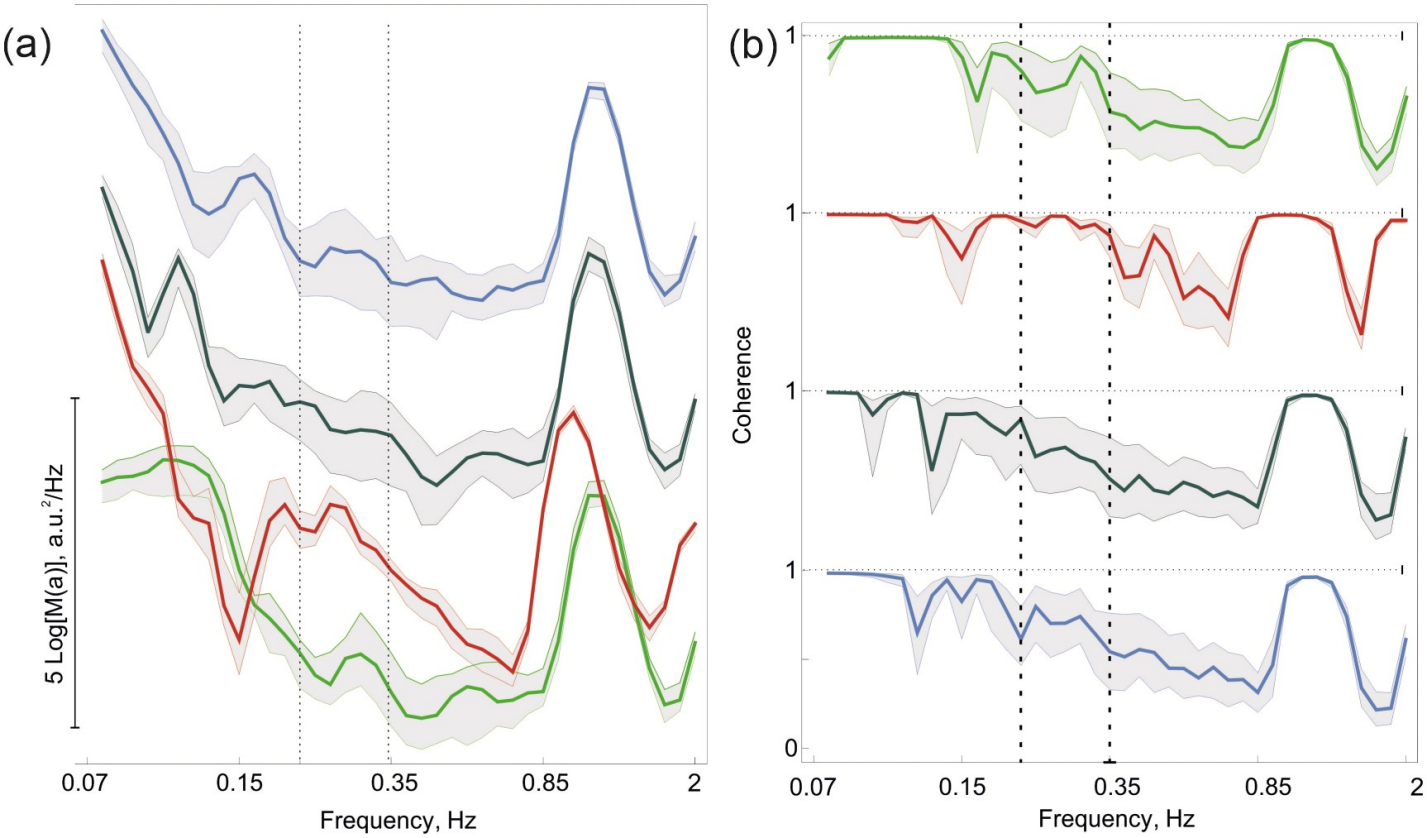
Spectra (a) and coherence (b) in 4 time intervals. Green curves—LSCI perfusion before the **BHT**, red—during the test, gray—transitional period, blue—recovered perfusion 1.5 min after the **BHT**. The color scheme is the same as in Fig 5.

The results of the wavelet spectra calculation are presented in Fig 6(a), the colour scheme is the same as in Fig 5. The coloured curves show the median values of spectral energy at every frequency *ν*, gray filled regions, limit 1/4 and 3/4 quantilies. Note that the spectra are shifted (2 units) from one another in the vertical direction. By comparing the curves, we found that the averaged spectral characteristics are very close, yet their peculiarities are of high research interest. Our study is focused on the respiratory influence of the perfusion spatial characteristics, and therefore the frequency under study is shown by two vertical dashed lines.

It is seen that the cardiac frequency during the **BHT** manoeuvre is lower than in **SR**; after returning to spontaneous respiration, the heart rate recovers. Another interesting feature is a decrease in the distance between the 1 and 3 quartiles in the respiratory frequency band during the **BHT**. This value characterises the divergence of the spectral characteristics.

In Fig 6(b), the averaged wavelet coherence is presented for four time intervals. The median value of the wavelet coherence of perfusion oscillations in the respiratory frequency band is higher in the **BHT** than in all other three time intervals. The difference is significant, which was confirmed by the Wilcoxon pair test. We assume that the key question in the validation of such coherency is if it is caused by the internal autocorrelation of the signals or by signal-to-signal phase relation. To clarify this item, the additional calculations based on surrogates were carried out. We utilized the surrogate technique for *short* data described in the Section Methods and estimated *Q* value (see Eq 8). The total number of the considered pairs was 20, and 100 surrogates were constructed for every temporal data. In the first time interval, only 2 pairs (*Q* = 10%) had significant correlation via surrogate testing. During **BHT**, 18 pairs were significantly coherent (*Q* = 90%), in the first recovery period, 12 pairs *Q* = 60% were significantly coherent, and 1.5 min later *Q* = 10% of the considered pairs were coherent.

Thus, we conclude that the **BHT** dramatically changes the phase relationship between the perfusion oscillations (around 0.3 Hz) distributed on the surface. During spontaneous breathing, a rather complicated behaviour is observed; the oscillations of approximately 0.3 Hz frequency are not coherent. During the **BHT**, these oscillations appear ordered and synchronised on the surface.

### Venous occlusion test

**Fig 7:**
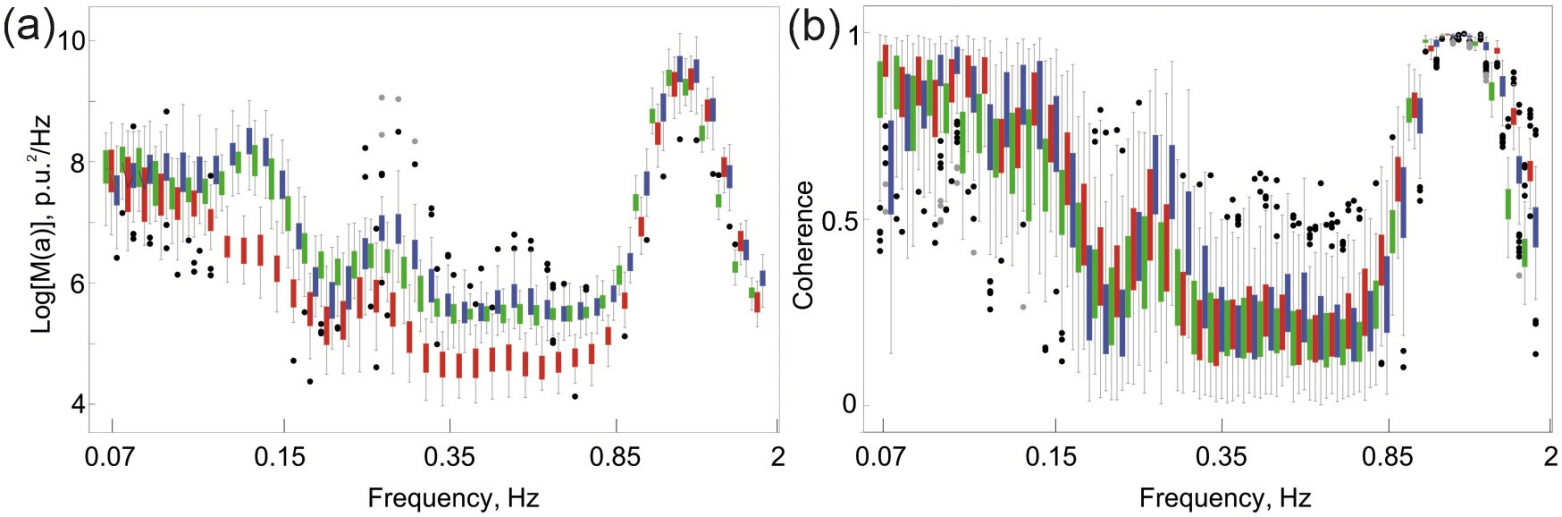
Averaged spectra (a) and coherence (b) of LSCI perfusion curves obtained at different *roi* during the VOT; green colour indicates data obtained during the SR, red—during the VOT, blue—during the SR after the VOT. Dots indicate outliers.

The approach developed for the two respiratory tests was extended to the **VOT**. Fig 7(a) presents the averaged spectral plot, where the spectra obtained before the test are shown by green rectangles, during the **VOT** by red, and after the removal of venous occlusion by blue.

The **VOT** significantly reduces the cutaneous blood flow. This fact can be attributed to a decrease in the blood velocity during venous stasis. Analysis of the spectra dynamics indicates that the venous occlusion is responsible for the reduction of the 0.1 Hz oscillations.

Furthermore, in spite of a significant reduction in the energy of 0.3 Hz oscillations, the peak remains well pronounced. After the removal of the pressure effect, the spectral properties of blood flow oscillations are fully recovered; the 0.3 Hz oscillations become stronger and have significantly higher phase coherence Fig 7(b).

## Discussion

LSCI provides a sequence of images of blood perfusion with appropriate spatial and temporal resolution [14]. This allows one to evaluate the spatial distribution of perfusion oscillations. On the one hand, the LSCI data gives very rich experimental data which can be considered in the context of blood flow coherency, but, on the other hand, analysis of the obtained 3D (time and two spatial dimensions) arrays is a very challenging task. In our previous work [20], we discovered the specific behaviour of the respiratory-related oscillations, which is a factor that motivates us to perform the research aimed at finding physiological tests that can influence the respiratory modulation.

At present, most researchers suggest that cutaneous blood flow pulsations of frequency around 0.3 Hz are associated with a venous breathing pump and that the sympathetic nerve activity can cause oscillations in a wide frequency range, overlapping the respiratory frequency band. It is worth noting that the role of respiratory-related oscillations is unique because they find their origin in a microvascular net, the venous part of the circulation system. The oscillations in the microvessels (the frequency of heart beat) occur due to the pulse wave propagation. The 0.1 Hz blood flow oscillations are caused by arteriolar tone variation and precapillary sphincters. Therefore, both of them occur because of the arterial part of circulation. One of the possible causes of unexpected spatial phase desynchronization of oscillations in the 0.14–0.6 Hz frequency band is the nature of their origin.

In this work, we have developed an experimental setup, data processing algorithms, and a set of physiological tests to support our hypothesis of the heterogeneity of respiratory-related oscillations. This pilot study included only one subject and three different research protocols. The study has limitations related to the lack of heart and respiratory rate monitoring and vasculature biopsy data. In the future, these protocols will be applied to a larger number of subjects.

We have found that the controlled respiration with a certain frequency increases the oscillation energy in the 0.14–0.6 Hz frequency band and significantly increases the phase coherence of such oscillations at the surface. Along with the fact that the heart rate decreases during the **BHT**, we have also established that this test dramatically changes the perfusion curve calculated over the *roi*, which size is close to the size of the microcirculatory unit supplied by one ascending arteriole [48, 53]. The **BHT** induces perfusion decrease which is caused by vasoconstriction. Very exciting results have been obtained in the measurements of the spatial structure of blood flow oscillations during the **BHT**. Visual inspection of the LSCI temporal dynamics shows that during spontaneous respiration the shape of the cardiac wave varies from *roi* to *roi*. Breathing holder induces variation in the form of the cardiac wave, especially in its diastolic part and makes oscillations more regular. This fact is illustrated by a decrease in spectral energy divergence in the defined frequency band at different *roi* and an increase in phase coherence of such oscillations during the **BHT**. The advantage of the approach proposed here is that we have verified the coherence under study by surrogate testing. Analysis of the results has revealed that, under relaxed conditions, the coherency of the oscillations with a frequency of approximately 0.3 Hz, which comprises only 20% of that of the randomly chosen pairs, does not exceed the significance threshold, whereas during the **BHT** the number of pairs with significant coherence is 80%.

In our opinion, the **BHT** is the most promising technique because it gives vivid results and is not very time-consuming. The main disadvantage of the **BHT** is its poor statistics due to time limitation, the maximum available duration for this test does not exceed 20–25 s and the number of independent oscillations does not exceed 5–7 cycles. We suppose that the proposed algorithms can partly overcome this deficiency.

The venous occlusion completely blocks the breathing pump—blood venous return to the heart from the periphery. Nevertheless, we have revealed that even during the **VOT** the cutaneous oscillations with the frequency of about 0.3 Hz exist, yet their energy becomes weaker. Therefore, we hypothesise that the cutaneous blood flow oscillations of 0.14–0.6 Hz are originated not only from the venous part of the circulation but can be produced by other mechanisms. One of the probable reasons is sympathetic nerve activity because it affects in the same frequency band.

Another interesting fact concerns the behaviour of around 0.3 Hz pulsations after the occlusion is removed. The energy of these pulsations significantly increases as much as the spatial coherence does. To illustrate the case, let us compare two states: before the **VOT** and after removal of the load; a significant increase in cardiac coherence is observed.

The obtained decrease of myogenic oscillations of the vascular tone (around 0.1 Hz) during **VOT** lays outside of the main line of the paper. It represents the known fact of myogenic oscillations decreasing in time of venous stasis [54]. It was shown that the venoarteriolar response may be due to the myogenic mechanisms associated with changes in vascular pressure, or it is mediated by a non-adrenergic, but neurally mediated, response [55].

One of the drawbacks of LSCI is that it leads to a large amount of data [56] which leads to a long process of analysis. Coherency testing analysis becomes very laborious and the development of testing algorithms for such data array is of particular importance.

All the obtained results support our hypothesis that the spatial coherence varies because of the tests selected. In addition, the increase in coherence was confirmed by the available statistical tests and through comparison with surrogate data. It is known that weak chaos is typical for the whole cardiovascular system [52] and particularly for the microvascular system [57]. This leads to a spatial complex behaviour of blood flow oscillations. It is interesting that it was found only for oscillations of around 0.3 Hz, which can be caused by the unique mechanism of respiratory-related oscillations origin.

Currently, the question whether the high-frequency flux motion is due to respiration or to vasomotion activity [5] is under discussion. Even though extensive research in this field began already in 1993, this question still requires an answer. We assume that the approach proposed in our study will help to elucidate this problem and will find application in other measurement techniques, in particular, in LDF imaging or photoplethysmography imaging.
